# The prognostic impact of macroscopic serosal change on resectable advanced gastric cancer

**DOI:** 10.1186/s12885-021-08767-8

**Published:** 2021-09-25

**Authors:** Masahiro Yura, Takaki Yoshikawa, Takeyuki Wada, Sho Otsuki, Tsutomu Hayashi, Yukinori Yamagata, Hitoshi Katai, Toshirou Nishida

**Affiliations:** Department of Gastric Surgery, National Cancer Center Hospital, 5-1-1 Tsukiji Chuo-ku, Tokyo, 104-0045 Japan

**Keywords:** Macroscopic serosal change, Gastric cancer, Prognostic factor

## Abstract

**Background:**

Advanced gastric cancer sometimes causes macroscopic serosal change (MSC) due to direct invasion or inflammation. However, the prognostic significance of MSC remains unclear.

**Methods:**

A total of 1410 patients who had been diagnosed with deeper-than-pathological-T2 gastric cancer and undergone R0 gastrectomy with lymph node dissection at the National Cancer Center Hospital during January 2000 and December 2012 were restrospectively reviewed.

**Results:**

MSC was not found in 108 of the 506 patients with pathological T4a (21.3%), whereas it was detected in 250 of the 904 patients with pathological T2-T3 (27.7%). The sensitivity, specificity and accuracy for diagnosing pathological serosa exposed (SE) by MSC were 78.7, 72.3 and 74.6%, respectively. The MSC-positive cases had a worse 5-year overall survival (OS) than the MSC-negative cases in pT3 (72.9% vs. 84.3%, *p* = 0.001), pT4a (56.2% vs. 73.4%, *p* = 0.001), pStageIIB (76.0% vs. 88.4%, *p* = 0.005), pStageIIIA (63.4% vs. 75.6%, *p* = 0.019), pStageIIIB (53.6% vs. 69.2%, *p* = 0.029) and pStage IIIC (27.6% vs. 50.0%, *p* = 0.062). A multivariate analysis showed that MSC was a significant independent predictor for the OS (hazard ratio [HR]: 1.587, 95%CI 1.209–2.083, *p* = 0.001) along with the tumor depth (HR: 7.742, 95%CI: 2.935–20.421, *p* < 0.001), nodal status (HR:5.783, 95% CI 3.985–8.391, *p* < 0.001) and age (HR:2.382, 95%CI: 1.918–2.957, *p* < 0.001). Peritoneal recurrence rates were higher in the MSC-positive cases than in the MSC-negative cases at each pT stage.

**Conclusions:**

In this study, the MSC was one of the independent prognostic factors in patients with resectable locally advanced gastric cancer.

## Background

Gastric cancer is one of the leading causes of cancer-related death worldwide [1]. Generally, the Union for International Cancer Control (UICC) tumour-node-metastasis (TNM) classification [2], which consists of the depth of tumor invasion, number of regional nodal metastasis and distant metastasis, is the standard staging system of gastric cancer. The accurate determination of the invasive depth and lymph node metastasis and the optimization of the pT and pN categories are critical for determining the extent of disease, guiding treatment planning and predicting outcomes [3]. Pathological serosal invasion is a particularly important prognostic factor in gastric cancer [4], as tumor cells exposed to the serosa can easily spread to the peritoneal cavity. The peritoneum is the most frequent site of distant metastasis in gastric cancer.

During operations, surgeons can diagnose tumor invasion exposed to the serosa based on changes in the color or irregularities at the serosal surface of the primary tumor. Such macroscopic serosal change (MSC) is usually consistent with pathological serosa exposed (SE), although it is sometimes indicative of pathological subserosa (SS). Conversely, macroscopic SS sometimes transforms to pathological SE. MSC is therefore related to the tumor progression but might reflect different reactions, such as inflammation.

Several previous studies have shown that patients with macroscopic serosal invasion had a worse prognosis than those without such invasion [5, 6]. However, they only showed that patients with pathologically negative but surgically positive serosal invasion had a similar survival to those with pathologically positive serosal invasion. They did not examine the recurrence pattern or deeply discuss the role of MSC. Furthermore, those studies ignored the combination of such findings with nodal metastasis when evaluating the survival impact of MSC. The prognostic value must be evaluated after adjusting for other key prognosticators in a multivariate analysis or by stratifying by the same TNM stage. Bando et al. [7] also reported that macroscopic serosal changes predicts peritoneal recurrence of gastric cancer. However, their study has only about one-third the number of patients compared to our study, but also includes D3 dissection, and includes about 32% of R2 resections.

Given the above, we examined the prognostic impact of MSC using latest TNM classification with a focus on peritoneal recurrence in patients with locally advanced gastric cancer which could be radical resection.

## Methods

### Study design

Retrospective observational study.

### Setting and participants

**Fig. 1:**
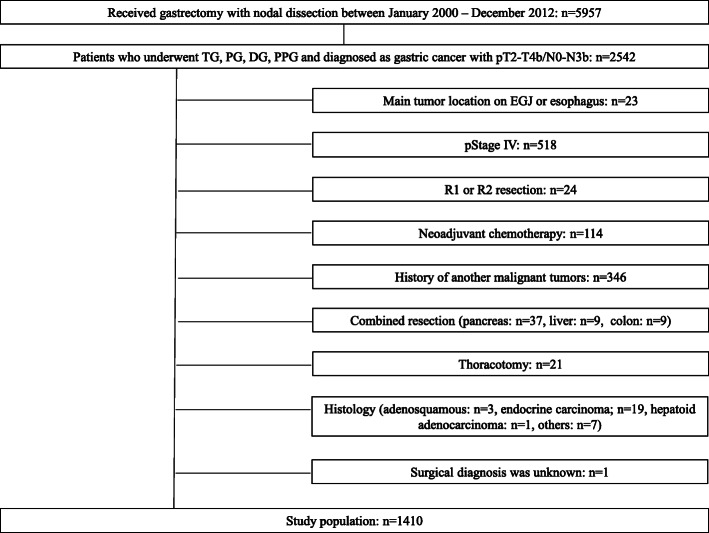
Study flow for the 5957 patients who underwent gastrectomy for gastric cancer between January 2000 and December 2012

All patients who received gastrectomy at the Department of Gastric Surgery, National Cancer Center Hospital during January 2000 and December 2012 were restrospectively reviewed. A total of 5957 patients underwent gastrecomty with lymph node dissection for primary gastric cancer. We selected the patients according to the following criteria; 1) underwent total, proximal, distal, or pylorus-preserving gastrectomy (TG/PG/DG/PPG) and 2) having primary gastric cancer of pT2-T4b/N0-N3b. The exclusion criteria were 1) main tumor located at the esophagogastric junction or esophagus, 2) a final diagnosis of stage IV (positive for peritoneal lavage cytology, para-aortic lymph node metastasis, or peritoneal dissemination), 3) R1 or R2 resection, 4) a history of neoadjuvant chemotherapy, 5) a history of other malignant disease, 6) received other organ resection except splenectomy for nodal dissection of the primary tumor, 7) received thoracotomy for the primary tumor, 8) tumors diagnosed with special pathological type (such as adenosquamous carcinoma; *n* = 3, endocrine carcinoma; *n* = 19, hepatoid adenocarcinoma; *n* = 1, others; *n* = 7) and 9) unknown intraoperative MSC. The flow diagram of the patients registered for this study is shown in Fig. 1.

Follow-up was conducted until death or for 5 years after surgery, whichever came first.

### MSC judgement

**Fig. 2:**
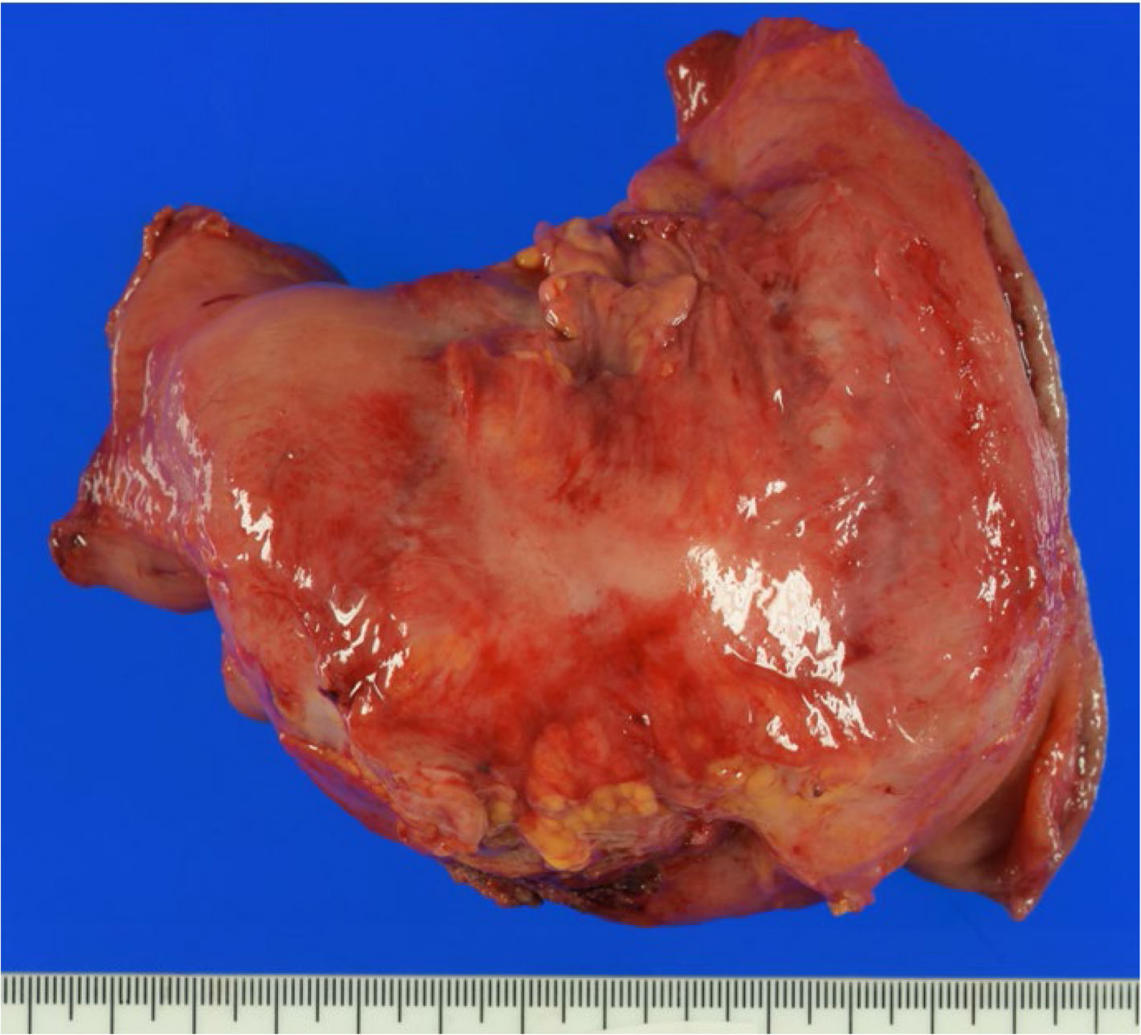
An example of the picture showing serosal surface of the distal stomach. The serosal surface of the tumor was hard and white in the center surrounded by the redness area. The tumor was judged as MSC+ but pathological invasion depth was T3

After resected the stomach, one experienced gastric surgeon checked the changes of the serosal surface matched to the tumor by visual inspection and palpation. Then, MSC was judged as positive when (1) the serosal color was changed to redness and/or whiteness and/or (2) the serosal surface was rough and/or hard. Figure 2 shows an example of the picture showing serosal surface of the distal stomach. The serosal surface of the tumor was hard and white in the center surrounded by the redness area possibly caused by macroscopic inflammation. The tumor was judged as MSC+ but pathological invasion depth was T3.

### Postoperative therapy and follow-up

Postoperatively, S-1 adjuvant chemotherapy was principally administered when a tumor stage was pStageII or III according to ACTS-GC criteria [8] after 2007. Physical examination and blood check including CEA and CA19–9 were checked every 3 to 6 months for the first 2 years postoperatively. Computed tomography was done every 6 months for the first 3 years and then every year for up to 5 years after surgery.

### Clinical and pathological factors

Progression of the tumor was determined by the 8th edition of the Union for International Cancer Control (UICC) tumor-node-metastasis classification of gastric carcinoma [2]. Background clinical and pathological factors included age, sex, surgical procedure, splenectomy (yes/no), main location of the tumor, maximum tumor diameter, macroscopic type according to the Borrmann classification, histological type, intraoperative macroscopic serosal change (+/−), pathological T factor, pathological N factor, pathological stage, and adjuvant chemotherapy (yes/no). The histopathological diagnosis was classified according to the 15th edition of the Japanese Classification of Gastric Carcinoma [9].

### Statistical analyses

All statistical calculations were done by SPSS statistical software program (ver. 24; SPSS Inc., Chicago, IL, USA). Group data were analyzed using the Pearson chi-squared test for categorical variables and the two-tailed Student’s t-test for continuous variables. OS was defined as the time between the date of surgery and the date of death due to any cause. Survival data were retrieved from hospital records. The OS were estimated using Kaplan-Meier curves and were compared by the log-rank test. A multivariate cox proportional hazards regression model was used to analyze the independent prognosis predictors. A *P* value of 0.05 was defined to evaluate statistical significance.

This study was conducted with the approval of the National Cancer Center Institutional Review Board (No. 2017–077).

## Results

### Patients

Among the 5957 patients who underwent surgery during the study period, 1410 patients who met the entry criteria were enrolled in this study. The median follow-up period was 78.0 months (range: 1–197 months). The background characteristics and pathological findings were shown in Table 1.MSC-positive tumors had more advanced stage than MSC-negative tumors, thus total gastrectomy and splenectomy were more frequently selected in patients with MSC-positive tumors than those with MSC-negative disease.

**Table 1:** Background characteristics and pathological findings of the patients

Characteristic	Total *n*=1410	MSC-positive *n*=648	MSC-negative *n*=762	*p*-value
Age	62.4+12.2	62.8+12.0	62.1+12.3	0.279
Sex				0.975
Male	958 (67.9)	440 (67.9)	518 (68.0)	
Female	452 (32.1)	208(32.1)	244 (32.0)	
Surgical procedures				<0.001
TG	553 (39.2)	311 (48.0)	242 (31.8)	
DG	764 (54.2)	334 (51.5)	430 (56.4)	
PG	18 (1.3)	2 (0.3)	16 (2.1)	
PPG	75 (5.3)	1 (0.2)	74 (9.7)	
Splenectomy				<0.001
Yes	354 (25.1)	241 (37.2)	113 (14.8)	
No	1056 (74.9)	407 (62.8)	649 (85.2)	
Main locatoin of the tumor				0.019
U	340 (24.1)	157 (24.2)	183 (24.0)	
M	620 (44.0)	253 (39.0)	367 (48.2)	
L	445 (31.6)	234 (36.1)	211 (27.7)	
Whole	5 (0.3)	4 (0.6)	1 (0.1)	
Tumor diameter (mm)	63.1 (37.5)	75.5 (40.5)	52.5 (31.1)	<0.001
Macroscopic type				<0.001
0	478 (33.9)	52 (8.0)	426 (55.9)	
I	64 (4.5)	27 (4.2)	37 (4.9)	
II	365 (25.9)	233 (36.0)	132 (17.3)	
III	371 (26.3)	246 (38.0)	125 (16.4)	
IV	100 (7.1)	78 (12.0)	22 (2.9)	
V	32 (2.3)	12 (1.8)	20 (2.6)	
Histological type				0.182
Differenteitated	517 (36.7)	225 (34.7)	292 (38.3)	
Undifferentiated	893 (63.3)	423 (65.3)	470 (61.7)	
UICC 8th				
Tumor invasion				<0.001
T2 (muscuralis)	412 (29.2)	33 (5.1)	379 (49.7)	
T3 (sub serosa)	492 (34.9)	217 (33.5)	275 (36.1)	
T4a (serosa exposed)	499 (35.4)	391 (60.3)	108 (14.2)	
T4b (serosa infiltrating)	7 (0.5)	7 (1.1)	0	
Pathological N factor				<0.001
N0	529 (37.5)	164 (25.3)	365 (47.9)	
N1	287 (20.4)	121 (18.7)	166 (21.8)	
N2	266 (18.9)	141 (21.8)	125 (16.4)	
N3a	228 (16.2)	144 (22.2)	84 (11.0)	
N3b	100 (7.1)	78 (12.0)	22 (2.9)	
Pathological stage				<0.001
StageIB	219 (15.5)	14 (2.2)	205 (26.9)	
StageIIA	263 (18.7)	55 (8.5)	208 (27.3)	
StageIIB	309 (21.9)	158 (24.4)	151 (19.8)	
StageIIIA	322 (22.8)	201 (31.0)	121 (15.9)	
StageIIIB	197 (14.0)	140 (21.6)	57 (7.5)	
StageIIIC	100 (7.1)	80 (12.3)	20 (2.6)	
Adjuvant chemotherapy				
Yes	353 (25.0)	220 (34.0)	133 (17.5)	<0.001
No	1055 (74.8)	427 (65.9)	628 (82.4)	
Unknown	2 (0.1)	1 (0.1)	1 (0.1)	

*TG* total gastrectomy, *DG* distal gastrectomy, *PG* proximal gastrectomy, *PPG* pylorus preserving gastrectomy, *MSC* macroscopic serosal change, *UICC* Union for International Cancer Control

### Accuracy of the macroscopic diagnosis of serosal invasion

Table 2 shows the relationship between the intraoperative macroscopic diagnosis and pathological diagnosis. MSC was not found in 108 of the 506 patients who were diagnosed with pathological T4a or T4b (21.3%), whereas it was detected in 250 of the 904 patients who were diagnosed with pathological T2-T3 disease (27.7%). The sensitivity, specificity and accuracy for diagnosing pathological SE based on MSC were 78.7, 72.3 and 74.6%. The proportions of overestimation (MSC-positive but pathological T2 or T3) were 5.1% (33 of 648) in pT2 and 33.4% (217 of 648) in pT3. The proportions of underestimation (MSC-negative but pathological T4a or T4b) were 14.2% (108 of 762) in pT4a and 0% (0 of 762) in pT4b.

**Table 2:** Intraoperative and pathological diagnosis of depth of tumor invasion

		MSC (+)		MSC (-)			Total
		sT4b	sT4a	sT3	sT2	sT1	
pSE(+)	pT4b	4	3	0	0	0	7
	pT4a	16	375	82	20	6	499
pSE(-)	pT3	8	209	146	77	52	492
	pT2	1	32	135	131	113	412
Total		29	619	363	228	171	1410

*MSC* macroscopic serocal change

### The overall survival

**Fig. 3:**
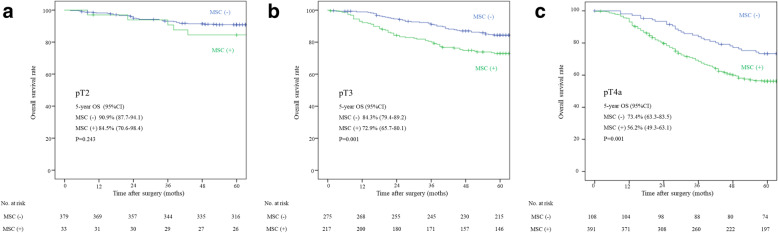
The 5-year survival rate in pT2–T4a according to MSC positivity. **A** The 5-year survival rates were slightly lower in patients with MSC (+) than MSC (−) T2 tumors (84.5% vs. 90.9%, *P* = 0.243). **B** and **C** The 5-year survival rates were significantly lower in patients with MSC (+) than MSC (−) tumors of pT3 (72.9% vs. 84.3%, *P* = 0.001) and pT4 (56.2% vs. 73.4%, *P* = 0.001)

**Fig. 4:**
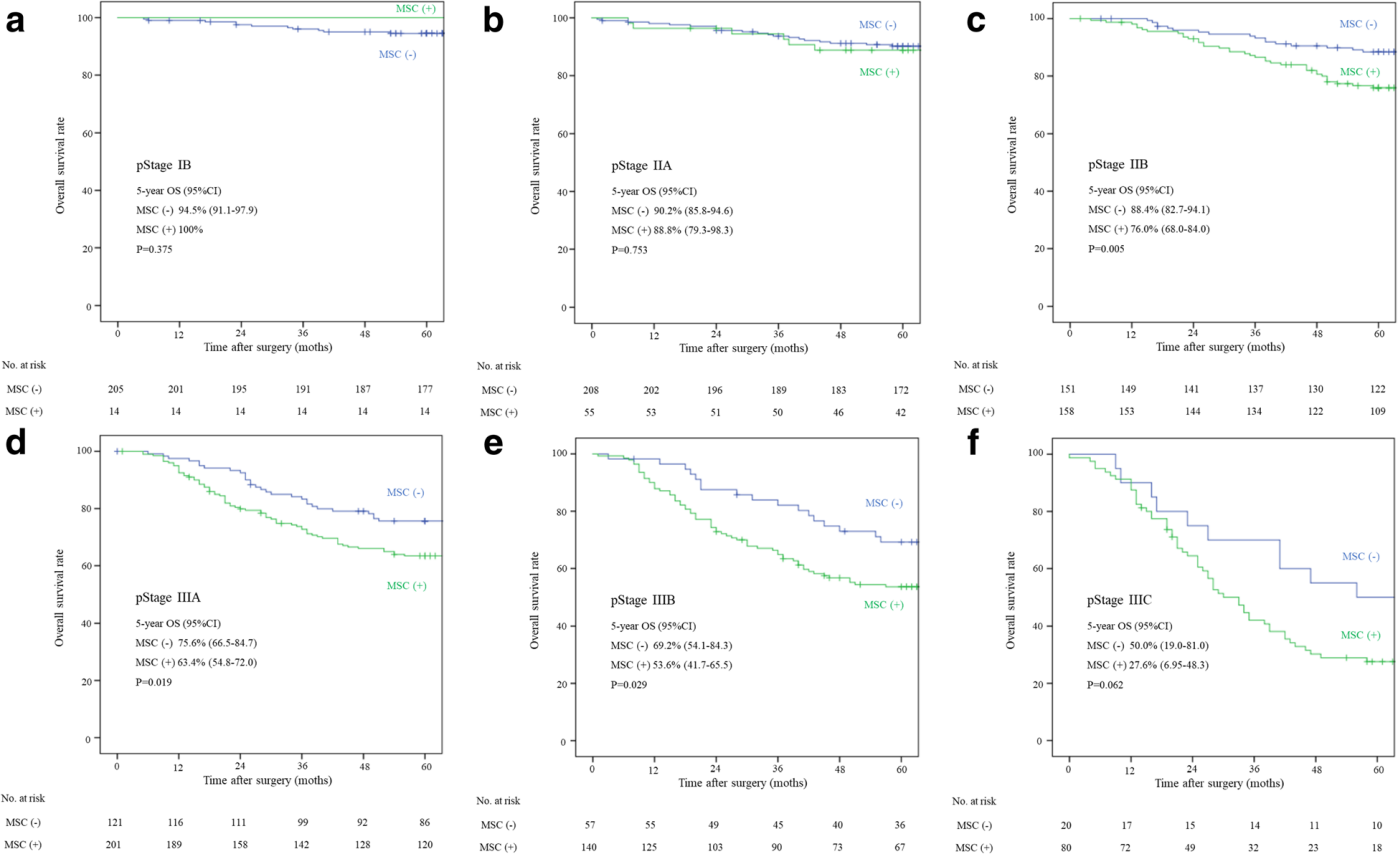
The 5-year survival rate in pStageIB–IIIC according to MSC positivity. **A** and **B** The 5-year survival rates were similar in patients with MSC (+) than MSC (−) tumors of pStageIB and IIA. **C**-**F** The 5-year survival rates were lower in patients with MSC (+) than MSC (−) tumors of pStageIIB (76.0% vs. 88.4%, *P* = 0.005), pStageIIIA (63.4% vs. 75.6%, *P* = 0.019), pStageIIIB (41.7% vs. 69.2%, *P* = 0.029) and pStageIIIC (27.6% vs. 50.0%, *P* = 0.062)

The overall survival (OS) was lower in MSC (+) patients than in MSC (−) patients at all T-stages, and the difference was significant in pT3 and pT4a (Fig. 3). The OS was also inferior in MSC (+) patients compared with MSC (−) patients in pStage IIB-IIIC (Fig. 4) to a significant degree, except for pStage IIIC.

A univariate analysis showed that the MSC, age, tumor diameter, tumor depth, nodal status, lymphatic invasion and vascular invasion were significantly associated with the OS. In the multivariate analysis, the MSC, age, pathological tumor depth and nodal status remained statistically significant (Table 3).

**Table 3:** Univariate and multivariate Cox proportional hazards analysis of clinicopathological factors

	Univariate			Multivariate		
Variables	HR	95% CI	*p*-value	HR	95% CI	*p*-value
Age						
<70	1.000			1.000		
>70	2.182	1.761-2.704	<0.001	2.382	1.918-2.957	<0.001
Tumor diamter (mm)						
<80	1.000					
>80	2.153	1.729-2.682	<0.001			
Histological type						
Differentiated	1.000					
Undifferentiated	0.642	0.762-1.183	0.642			
Macroscopic serosal change (MSC)						
MSC negative	1.000			1.000		
MSC positive	3.107	2.466-3.914	<0.001	1.587	1.209-2.083	0.001
UICC 8th						
Tumor invasion						
pT2	1.000			1.000		
pT3	2.276	1.572-3.296	<0.001	1.464	0.989-2.168	0.057
pT4a	4.934	3.497-6.960	<0.001	2.505	1.672-3.755	<0.001
pT4b	13.878	5.464-35.247	<0.001	7.742	2.935-20.421	<0.001
Pathological N factor						
pN0	1.000			1.000		
pN1	1.988	1.375-2.875	<0.001	1.776	1.225-2.575	0.002
pN2	2.843	2.003-4.036	<0.001	2.576	1.800-3.661	<0.001
pN3a	4.573	3.275-6.386	<0.001	3.464	2.462-4.873	<0.001
pN3b	9.385	6.556-13.434	<0.001	5.783	3.985-8.391	<0.001
Lymphatic invasion						
Negative	1.000					
Positive	2.131	1.640-2.768	<0.001			
Vascular invasion						
Negative	1.000					
Positive	1.657	1.214-2.235	0.001			
Adjuvant chemotherapy						
No	1.000					
Yes	0.942	0.777-1.265	0.991			

*MSC* macroscopic serosal change, *UICC* Union for International Cancer Control

### Recurrence patterns

There were significant differences in the rate of recurrence between MSC (+) and MSC (−) patients (40.6% vs. 14.1%, respectively; *p* < 0.001). The most predominant site was the peritoneum, followed by the lymph nodes in both groups. Peritoneal recurrence rates were higher in the MSC (+) group than in the MSC (−) group at each pT stage (Table 4), with statistical significance noted for pT2 (9.1% vs. 0.5%; *p* = 0.004) and pT4a/b (30.7% vs. 16.9%; *p* = 0.015).

**Table 4:** Relationship between peritoneal recurrence and macroscopic serosal change (MSC)

Peritoneal recurrence	MSC negative			MSC positive		
	pT2	pT3	pT4a/4b	pT2	pT3	pT4a/4b
Positive	2	13	20	3	17	122
Negative	377	261	98	30	200	276
Total	379	274	118	33	217	398
Rate of peritoneal recurrence (%)	0.5%	4.7%	16.9%	9.1%	7.8%	30.7%

*MSC* macroscopic serosal change

## Discussion

We explored the prognostic significance of MSC in patients with advanced gastric cancer. Even after stratification by T stage or final stage using the Eighth TNM Classification, the patients with MSC had a poorer prognosis than the patients without MSC, except for those with early stage disease. Furthermore, MSC was an independent prognostic factor for OS. These results suggest that MSC can be used to further stratify patients of identical T stage and final TNM stage. Therefore, MSC has utility for predicting the prognosis of patients with advanced gastric cancer.

Several studies have shown that MSC has a poor prognosis. Wang et al. [10] reported that pT3 MSC (+) patients had a similar prognosis to pT4a patients. Sang et al. [6] showed that pT2–3 MSC (+) patients had a similar survival rate to pT4a MSC (−) patients. However, in those studies, the effects of MSC on the final pathological stage, the combination of T stage and lymph node metastasis, were not evaluated. Bando et al. [7] reported that the magnitude of serosal changes predicted peritoneal recurrence of gastric cancer; they also showed that pT2 patients with marked macroscopic serosal invasion had a poorer prognosis than pT3 and pT4 patients with little or no macroscopic serosal invasion. However, that study involved only about one-third the number of patients as compared to our present study. In addition, this study also included patients who underwent D3 (para-aortic) lymph node dissection, and the R2 resection rate was 32%. In contrast, our study involved strict eligibility to accurately verify the impact of MSC on the patients with resectable advanced gastric cancer.

Intraoperative MSC is determined based on the color of, and morphological differences between, the tumoral and adjacent normal surface of the serosa. In this study, MSC was found in pT2 and pT3 tumors, suggesting that it reflects not only the tumor itself but also reactions to it, such as inflammation. In pT4 cases, MSC may be negative when the tumor shows only slight invasion of the serosa without inflammation. Therefore, MSC-positive cases may have either a substantial tumor volume at the serosal surface or accompanying inflammation. Recently, it has been known that inflammatory reactions play important roles in the growth of tumors. Both cancer cells and the surrounding stromal and inflammatory cells engage in well-orchestrated reciprocal interactions to form an inflammatory tumor microenvironment that promotes tumor growth, angiogenesis, and metastasis [11, 12]. Our study showed that the rate of peritoneal recurrence was higher in MSC-positive than -negative pT2–T4 patients with negative lavage cytology (Table 4). This means that tumor cells can directly invade the serosa or cause serosal changes indirectly, such as by inducing inflammation, which can in turn cause peritoneal changes at distant sites even if intraoperative lavage cytologic analysis yielded a negative result. Previous studies also showed that the invasion area of the tumors at the serosal surface, and the magnitude of serosal change, are risk factors for peritoneal metastasis and prognosis [7, 13].

Most important finding in our present study is that not only MSC is an independent prognostic factor, similar to pT and pN, but also that MSC further stratifies the prognosis in patients with late-stage gastric cancer after stratifying by final TNM stage. This may lead to more efficacious postoperative adjuvant therapies, including switching from single to dual agents. Also, confirmation of the presence of MSC by staging laparoscopy may influence the selection of treatments such as preoperative chemotherapy in the future.

On the other hand, there was no marked difference in survival between MSC (+) and MSC (−) pStage IB and IIA patients. The stage IB group included only T2N0 cases, and the stage IIA group included T3N0 and T2N1 cases. MSC was not associated with peritoneal metastasis when pT2 and pT3 patients had no nodal metastasis. Peritoneal dissemination is established by detachment cancer cells from the gastric serosa and attachment to, and growth at, the peritoneum. And another mechanism of peritoneal dissemination is the release of tumor cells via lymphatic channels [14]. Pathologically serosa-negative (T2 or T3) tumor is thought to occur peritoneal dissemination from tumors with some degree of lymph node metastases.

Although the Kaplan–Meier curves of MSC (+) and MSC (−) stage IIIC patients were clearly dissociable, the difference in OS was not significant. This is likely because of the small number of MSC (−) cases included in the stage IIIC group (*n* = 20). In other words, cases having pT3 or pT4 tumor with extensive lymph node metastasis (7 ≤ N) classified as pStage IIIC almost show MSC (+). Accurate verification of the impact of MSC on Stage IIIC will require further cases.

This study had several limitations. First, it used a retrospective design and included patients treated at only a single cancer center. Therefore, a multicenter, prospective study is required to validate the present results. Second limitation is that the MSC-positive rate may be different in other hospitals. Sang et al. [6] reported the diagnostic accuracy for MSC to be 82.1%, with 87.1% sensitivity and 81.1% specificity. Our results showed a diagnostic accuracy of 74.6%, sensitivity of 78.7% and specificity of 72.3%. Although the difference was not large, there are some discrepancies for diagnosing MSC, depending on the institution and country. Third, we speculate that MSCs without tumor invasion may be affected by inflammation around the tumor; however, we did not evaluate inflammatory reactions pathologically in this study. We will investigate whether MSCs are associated with inflammation pathologically in future studies.

## Conclusion

In this study, the MSC was one of the independent prognostic factors in patients with resectable locally advanced gastric cancer. Prospective validation study is necessary to confirm the present results.

## Data Availability

The datasets used and analyzed during the current study are principally not available.

## Abbreviations

MSC: Macroscopic serosal change
OS: Overall survival
SE: Serosa exposed
HR: Hazard ratio
UICC: Union for International Cancer Control
TNM: Tumor node metastasis
SS: Subserosa
TG: Total gastrectomy
DG: Distal gastrectomy
PG: Proximal gastrectomy
PPG: Pylorus preserving gastrectomy
